# Beyond Authoritarian Personality: The Culture-Inclusive Theory of Chinese Authoritarian Orientation

**DOI:** 10.3389/fpsyg.2016.00924

**Published:** 2016-06-30

**Authors:** Chin-Lung Chien

**Affiliations:** ^1^Psychology, Kaohsiung Medical UniversityKaohsiung, Taiwan; ^2^Positive Psychology Center, Kaohsiung Medical UniversityKaohsiung, Taiwan

**Keywords:** authoritarian orientation, authoritarian personality, Confucian relationalism, culture-inclusive theory, cultural system approach, indigenous psychology, Mandala Model of Self

## Abstract

In a dyad interaction, respecting and obeying those with high status (authority) is highly valued in Chinese societies. Regarding explicit behaviors, Chinese people usually show respect to and obey authority, which we call authoritarian orientation. Previous literature has indicated that Chinese people have a high degree of authoritarian personality, which was considered a national character. However, under Confucian relationalism (Hwang, 2012a), authoritarian orientation is basically an ethical issue, and thus, should not be reduced to the contention of authoritarian personality. Based on Yang's (1993) indigenous conceptualization, Chien (2013) took an emic bottom-up approach to construct an indigenous model of Chinese authoritarian orientation; it represents a “culture-inclusive theory.” However, Chien's model lacks the role of agency or intentionality. To resolve this issue and to achieve the epistemological goal of indigenous psychology (that is, “one mind, many mentalities”), this paper took the “cultural system approach” (Hwang, 2015b) to construct a culture-inclusive theory of authoritarian orientation in order to represent the universal mind of human beings as well as the mentalities of people in a particular culture. Two theories that reflect the universal mind, the “Face and Favor model” (Hwang, 1987) and the “Mandala Model of Self” (Hwang, 2011a,c), were used as analytical frameworks for interpreting Chien's original model. The process of constructing the culture-inclusive theory of authoritarian orientation may represent a paradigm for the construction of indigenous culture-inclusive theories while inspiring further development. Some future research directions are proposed herein.

## Introduction

In “My First Teacher,” Lu Xun, a well-known Chinese writer, stated, “In the center of our house, a memorial tablet was worshiped on which heaven (天), earth (地), emperor (君), parents (親), and teacher (師) were written in gold color. They represented the five figures we have to respect and obey.” In traditional Chinese society, emperor (ruler), parents and teacher were regarded as the supreme authorities comparable to heaven and earth. Chinese society had long been ethic-based (Liang, 1949; Hwang, 2012a). From birth to death, Chinese people are embedded in various interpersonal networks, including relationships between ruler-subordinate, father-son, husband-wife, older brother-younger brother, in addition to friend-friend. The hierarchies of relationships are particularly emphasized in Chinese society. Among the five cardinal relationships, four are vertical and those who occupy the superior roles are regarded as authorities (Chien, 2013). As the worshiped tablet in Lu's house demonstrates, Chinese people emphasized a reverence for, and obedience to, authorities (i.e., emperor, parents and teachers); authorities were even regarded as gods (heaven and earth).

The influence of Western culture has persisted for over 100 years; recently, globalization has been an inescapable trend. However, reverence for, and obedience to, authorities has never faded away in Chinese societies. American sinologist Wright (1962) listed 13 traits of traditional Chinese; the first was “obedience to authority (parents or superiors).” In a qualitative study (Chuang, 1987) 14 Chinese adults with different backgrounds were interviewed for their experiences in interacting with various authorities (e.g., parents); it showed that most participants revealed fear of and/or obedience to authorities. Furthermore, in a case study, Cheng (1995) found that, in a private enterprise, the subordinates usually complied with the boss's opinions without raising their own ideas in meetings. Such interaction patterns are quite distinct from those in Western cultures. Similar patterns were found in quantitative studies with large sample sizes. Chien and Huang (2010) found that that “reverence for teachers” and “obedience to teachers' instructions” were regarded as role obligations for students from elementary school to college. Huang and Chu (2012) found “obedience to superiors” and “filial piety” to be highly valued; “respecting superiors” was considered the most important value orientation. Also, Zhang et al. (2005) found “relational hierarchy” to be an important value in contemporary Chinese societies.

The aforementioned studies demonstrated that even under the trend of modernization or globalization, the tendency of respecting and obeying authorities, which we called “authoritarian orientation” (Yang, 1993; Chien, 2013), is still ubiquitous in contemporary Chinese societies. The modernization process of Chinese societies mainly takes that of Western societies as a paradigm. Chinese authoritarian orientation was regarded as one of the most negative characters that had to be removed from Chinese culture. As a result, it became the focal topic for many Chinese scholars (Yang, 1965; Li and Yang, 1972; Wei, 1974; King, 1979). Authoritarian personality (Adorno et al., 1950) was taken as the theoretical framework to interpret Chinese authoritarian orientation and was even considered the national character of the Chinese people (Wright, 1962; Yang, 1965; Li and Yang, 1972; King, 1979).

However, as the influence of Western mainstream psychology spread all over the world, psychologists from non-Western cultures began to reflect on whether the imported Western psychological knowledge and theories could be applied to the local context. Since the 1980s, an indigenous approach to psychology (indigenous psychology) has emerged in non-Western societies, representing a challenge to Western psychology (Allwood and Berry, 2006). At the same time, an increasing number of psychologists in Chinese societies, especially in Taiwan and Hong Kong, have committed to the development of Chinese indigenous psychology. Recently, much progress has been made on various topics (see Yang K.-S., 1999). As for Chinese authoritarian orientation, Yang (1993) offered preliminary descriptions. Only recently has a systematic model of authoritarian orientation been proposed from an indigenous emic approach (Chien, 2013; Chien and Huang, 2015). The formation process and psychological components of authoritarian orientation constructed by Chien (2013) illustrate why Chinese people revere and obey authority to a great extent; however, it cannot explain their resistance to authority in specific situations.

The main purpose of this paper is to construct a “culture-inclusive theory” of Chinese authoritarian orientation that can not only explain Chinese people's reverence for, and obedience to, authority but can also explain their resistance to authority. In order to have a context to follow, the theory is proposed in the process of theoretical progress. First, the theory of authoritarian personality suggested by Western psychology is presented and the reason why it fails to offer an appropriate understanding of Chinese authoritarian orientation is explicated. Second, Yang's (1993) preliminary conceptualization and Chien's (2013) model based on Yang (1993) are introduced. Third, to overcome the deficiencies in Chien's model, this paper goes a step further to propose a comprehensive culture-inclusive theory from a “cultural system approach” with supporting studies provided. Finally, the advantages of the new theory are addressed and future directions are discussed as well.

## The fallacy of the authoritarian personality

It has long been believed that the theories constructed by Western psychologists (mainly psychologists in North America) are universal. Before the emergence of Chinese indigenous psychology, the authoritarian personality was used as a framework for understanding Chinese authoritarian orientation. Although cross-cultural comparisons indicated that Chinese people had higher scores on authoritarian personality (measured by F scale) than did Westerners (Singh et al., 1962; Meade and Whittaker, 1967), these studies using the etic approach failed to reveal the cultural system and meanings behind Chinese people's tendencies to respect and obey authority.

### Theory of authoritarian personality

Western scholars' interest in the authoritarian personality originated from anti-Semitism. Adorno et al. (1950) claimed that German anti-Semitism could be attributed to their authoritarian personality, which was regarded as the potential psychological roots of anti-democratic tendencies and fascism. It is composed of a set of psychological syndromes including conventionalism, authoritarian submission, authoritarian aggression, anti-intraception, superstition and stereotypy, power and toughness, destructiveness and cynicism, projectivity, and concern with sex (Adorno et al., 1950; Brown, 1965). In addition, the authoritarian personality was seen as a significant predictor of ethnocentrism and prejudice toward outgroups, not restricted to Jews (Brown, 1965; Whitley, 1999).

Initially, the theory of the authoritarian personality was developed based on the theories of psychoanalysis, and it was hypothesized that the formation of an authoritarian personality originated from the experiences of interacting with authority figures (parents) since childhood (Brown, 1965; Sanford, 1973). Brown (1965) indicated that if children were treated strictly by authority figures or parents, they would probably hold hostile and aggressive tendencies toward authority figures. Since these inner psychological conflicts could not be expressed explicitly, they had to be transformed into socially acceptable forms through defense mechanisms. As a result, hostility and aggressiveness toward authority would be transformed into authoritarian personality syndromes. A recent cross-cultural study found that children's authoritarian disposition was fostered by authoritarian parenting (Kornyeyeva and Boehnke, 2013).

The launch of “The Authoritarian Personality” (Adorno et al., 1950) initiated a series of studies, discussions and criticisms since the 1950s (Stone et al., 1993). The theory has been challenged and modified many times (Altemeyer, 1998; Funke, 2005; Oesterreich, 2005), and keeps evolving (Duckitt, 2013). As Duckitt (2013) indicates, in the beginning, it was defined as a set of personality dimensions or a personality structure composed of psychological syndromes. Subsequently, it was regarded as a set of social attitudes or ideologies (right-wing authoritarianism, RWA). Recently, authoritarianism was further defined as the response patterns toward external threats. Among the various modifications, the conceptualization of RWA and its measures are perhaps the most investigated. Authoritarianism was reduced to three components: authoritarian submission, authoritarian aggression, and conventionalism (Altemeyer, 1998). It was used to predict political attitudes and behaviors, for example, American Whites' prejudice toward African Americans (Whitley, 1999).

### The gap between Western theory and chinese life world

Western psychological theories represent the scientific micro-world constructed and developed based on the life world of Western cultures. From the perspective of constructive realism, there is always a gap between micro-world and life world (Hwang, 2012b). Furthermore, life worlds across different cultures are quite divergent. As a result, if we use theories constructed based on the Western life world to explain Chinese psychological character and behaviors, there will not only be a gap, but rather an unbridgeable gap. This gap may also lead to the alienation between the local people and the discipline of psychology in non-Western cultures.

Based on sociology of knowledge, any theories in social science are bound by socio-cultural contexts (Hamilton, 1990; Yang, 2000). The conceptualization of the authoritarian personality was greatly affected by Jewish left-wing intellectuals (Shih, 1998). As researches progressed, the authoritarian personality was found to reflect right-wing authoritarianism instead of being neutral (Altemeyer, 1998). In short, the authoritarian personality was developed under specific social, cultural and historical contexts, and was also closely related to the ideologies of Western political development. Although the authoritarian personality is constantly evolving, it reflects an imposed etic approach for understanding Chinese people's tendency to respect and obey authorities, incompatible with Chinese cultural tradition.

In addition, the authoritarian personality is based on the assumption of individualism, specifically “methodological individualism,” which claims that individuals are the unit of social scientific analysis (Hwang, 2000). However, this assumption cannot be applied to Chinese culture based on relationalism (Ho, 1998; Hwang, 2012a). Chinese relationalism particularly emphasizes role ethics originating from relational proximity and hierarchy (Chuang, 1998; Hwang, 2012a). Thus, in Chinese societies, social interactions in vertical relationships entail special cultural meanings (Hwang, 1995), that is, role ethics in dyadic relationships. Most importantly, this is where the authoritarian personality cannot apply. In sum, the authoritarian personality is not an appropriate conceptualization to understand Chinese people's interactions with authorities.

### Calling for an indigenous theoretical model

As cultural influences have increasingly been highlighted in psychology, the assertion that psychological theories from North America are universal has been challenged. Currently, many psychologists claim that North American psychology is culture bound and is based on the assumption of individualism; as such, it can be said to be the indigenous psychology of North America (Yang, 2000; Allwood and Berry, 2006).

In order to reflect cultural influences, cross-cultural psychologists have developed various cultural dimensions to locate different societies around the world. Among these dimensions, individualism-collectivism (I/C) is the most representative one in explaining the differences among psychological characters and behaviors across different cultures (Oyserman et al., 2002). Chinese culture is regarded as characterized by vertical collectivism (Triandis and Gelfand, 1998) and high power distance (Hofstede, 2003). However, the I/C approach, also a “pan-cultural dimension” approach (Bond, 2015; Hwang, 2015b) has been criticized for its over-simplicity and vagueness (Fiske, 2002; Miller, 2002). Under such an approach, Chinese people's psychological characteristics can be understood only if they are described in contrast to Americans (Hwang, 2012a).

Some indigenous dimensions, such as hierarchical interdependence (Bond and Hwang, 1986), authority-directed (Lew, 1998), and hierarchical relationalism (Liu, 2015) are used to refer to Chinese culture by Chinese psychologists. Those labels enable people to form a rough impression of Chinese people. However, they fail to offer a systematic and detailed understanding of Chinese people's interactions with authorities. Therefore, an indigenous theoretical model is needed.

## Theoretical development of authoritarian orientation

After the emergence of Chinese indigenous psychology, Yang (1993) proposed the preliminary conceptualization of authoritarian orientation. Yang never explicitly stated that his conceptualization was meant to replace the authoritarian personality; however, it has much higher “indigenous compatibility” (Yang, 1997, 2000) than the authoritarian personality.

### Yang's preliminary conceptualization

Yang (1981, 1993, 1995) has worked on the concept of social orientation as a theoretical construct for describing and understanding Chinese people's personality and social behaviors. Chinese social orientation is composed of four sub-orientations: “familistic” (the Chinese version of group orientation), “relationship,” “authoritarian,” and “other” orientation. Authoritarian orientation was defined as the inclination of a subordinate to submit to, cooperate with, or merge into the authority figures. It consists of three parts: “Authority sensitization” signifies a social interaction; Chinese people always try to find out if there is any authority present so that they will know how to interact with another party. “Authority worship” means that Chinese people worship not only living authorities but also ancestors and historical heroes. In addition, authority worship is usually unconditional, without restrictions or limitation in scope and time. “Authority dependence” means that Chinese people see authorities as trustworthy and almighty so that they are completely dependent on those authorities. Moreover, they tend to show a kind of self-surrendering submissiveness and display a syndrome of psychological disability when facing an almighty authority. “Authority dread” was added as a new component by Yang (2004).

Traditionally, Chinese people made their living in an ecological environment suitable for intensive agriculture, which probably led to Chinese familism (Yang and Yeh, 2005). Under familism, most relationships in a family were vertical-based and the family head had the greatest authority. Authoritarian orientation was very likely generated in such a context (Yang, 1993). Through constant interactions with authority figures (e.g., parents and elders) in the family, Chinese people gradually acquired a set of authoritarian-oriented inclinations. People's repeated authoritarian- oriented interactions thus eventually resulted in certain authoritarian-oriented traits, which in turn inclined them to interact in an even more authoritarian-oriented way under identical or similar social conditions (Yang, 1993). Through the process of “pan-familization,” Chinese people also exhibited behaviors similar to authoritarian orientation when interacting with authorities outside the family.

### Model of Chinese authoritarian orientation: emic approach

Although Yang's (1993) conceptualization is highly compatible to the indigenous context, it only represents preliminary constructs that are more like a description or classification than a systematic framework. Based on Yang's conceptualization, Chien (2013) attempted to reconstruct authoritarian orientation as a systematic model.

#### Cultural ideals in vertical social interaction

Based on Confucian relationalism, Chinese culture can be regarded as a system structured mainly on the principles of “favoring the intimate” and “respecting the superior” (Hwang, 2012a). These principles represent the norms governing Chinese people's social interactions. Furthermore, the principle of “respecting the superior” represents the norm by which a subordinate should treat or serve a superior in hierarchical relationships (Hamilton, 1990; Hwang, 1995). Five relationships (the five cardinals) are particularly important in Confucian culture; they have corresponding role obligations which emphasize that a subordinate should obey and respect a superior.

“*Kindness on the part of the father, and filial duty on that of the son; gentleness on the part of the elder brother, and obedience on that of the younger brother; righteousness on the part of the husband, and submission on that of the wife; kindness on the part of elders, and deference on that of juniors; and benevolence on the part of the ruler, and loyalty on that of the minister.”* (Li Yun, Li Chi).

“*The love for one's parents is really humanity and the respect for one's elders is really righteousness. All that is necessary is to have these natural feelings applied to all men.”* (Jin Xin, part one).

**Figure 1:**
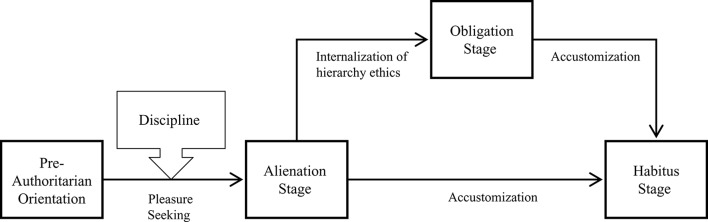
**The process of authoritarian orientation formation (adapted from Chien, 2013)**.

A famous scholar, Wei (1974), indicated that “not disobeying” and “respecting elders” were the core spirit of Chinese socialization. However, these cultural ideals are the discourses at the sociological level as well as at the sollen level. In order to examine how Chinese people interact with authorities in real life, which is a question at the practical level, empirical researches will have to be conducted (Hwang, 2012a). Chien (2013) conducted a qualitative study in which 18 participants (10 men and 8 women) were interviewed for their experiences of interacting with authority figures (e.g., parents and teachers) since childhood. The components and formation process of authoritarian orientation were constructed as a substantive theory of Chinese authoritarian orientation, as illustrated below (see Figure 1; Table 1).

**Table 1:** **Components of authoritarian orientation (adapted from Chien, 2013)**.

	**Stage**	**Components**	**Possible Actions**
1.	Pre-authoritarian	−	−
2.	Alienation	Authority-dread	Keeping a distance with fear
			Interacting with caution
			Submitting with self-suppression
		Authority-dependence	Conforming and coordinating
			Pleasing and ingratiating
3.	Obligation	Authority-reverence	Respecting heartfully
		Authority-obedience	Obeying sincerely
4.	Habitus	Authority-sensitization	Authoritarian-oriented Habitus (responding automatically and habitually)

#### Components and the formation process

At the inception of life, a naïve and uneducated self with instincts and impulses comes to the world. In the stage of *pre-authoritarian orientation*, the naïve self, also a biological individual, is tolerated with social indulgence. It then enters the cultural world that emphasizes role obligations, particularly for a subordinate to respect and obey the authorities, which we call Chinese “hierarchy ethics.” Social disciplines are implemented by socialization agents (e.g., parents) to shape, train and educate the biological individual to become a cultured person.

However, the naïve self and cultural demands are always in conflict to some extent. The agents usually utilize punishment and reward to force individuals to accept and practice these cultural values and norms. However, under such conditions, ethics will not be genuinely accepted but will probably be alienated to “authority-dread” and “authority-dependence” in the stage of *alienation orientation*. In addition, children are not completely passive in socialization. Social learning also plays an important role. For example, a child may fear an authority through observing that his sibling was strictly scolded by parents. For the alienation orientation, authority-dread is defined as an affective attitude that is based on fear and dread toward authorities. Authority-dependence is regarded as a desire for positive regards, affirmations and praise from authorities.

As one starts to internalize hierarchy ethics and accept the role obligations, “authority-reverence” and “authority-obedience” emerge as new components in the stage of *obligation orientation*. People behave respectfully and obey authorities due to the fact that hierarchy ethics has been internalized as their core values in accordance with Confucian cultural ideals. Compared with “teaching through words,” “teaching by personal example” is more important for the internalization of hierarchy ethics. Children are more likely to identify with their parents and model what parents do if children and parents have positive affection (Li, 1998). If they do not have a positive relationship, social learning will probably not happen as expected. Authority- reverence and authority-obedience are both defined as obligation-based beliefs reflecting appropriate interactions with authorities. The former refers to the cognition that a subordinate should behave respectfully in interactions with authorities; the latter refers to the belief that a subordinate should submit to authorities' opinions and commands.

When people repeatedly make authoritarian-oriented actions, these actions will become habitus. In the stage of *habitus orientation*, people acquire a set of authoritarian-oriented habitus. On a social occasion like a wedding reception or a conference coffee break, for example, people are accustomed to verifying whether an authority figure is nearby (*authority-searching*). Subsequently, they will automatically behave respectfully if an authority figure is present. In addition, they will automatically obey authority figures as well. This authoritarian-oriented habitus is named *authority-sensitization*. The automatization of authoritarian-oriented actions is the focal point in this stage. One may have learned authoritarian-oriented habitus before going through the obligation stage. However, theoretically, the habitus should be more stable after going the obligation stage.

The emic approach with bottom-up strategies is advocated by many indigenous psychologists. Chien (2013) also took this approach to construct the formation process and psychological components of Chinese authoritarian orientation. Authoritarian orientation involves the psychological characteristics and behavioral tendencies acquired through repeated social interactions. As a result, the authoritarian-oriented actions will be exhibited in different vertical interactions with authorities. This corresponds to the perspective of “person-situation interactions” (McAdams, 1997) and has high compatibility with Chinese relationalism.

### Problems to be solved

Instead of authoritarian personality, Chien (2013) elaborated on Yang's conceptualization and developed a systematic model with high indigenous compatibility. However, there were still problems to be solved. The formation process of authoritarian orientation begins with a non-authoritarian orientation and progresses to the final stage of habitus. Through the formation process, people (subordinates) are guided to be submissive toward authorities. That is, there seems to be no space for agency or intention of self in the model, although the model is never against human agency and intention. This is the first issue and the main problem to be solved in this paper.

Based on the principle “one mind, many mentalities” (Shweder et al., 1998), Hwang (2011b,c) advocated that the epistemological goal of indigenous psychology is to construct a series of “culture- inclusive theories” that represent not only the universal human mind determined by biological factors but also the particular mentality of people in a given culture (also see Hwang, 2015a,b). Although Chien's (2013) model is highly compatible with indigenous culture, the emic approach he took does not guarantee the achievement of the epistemological goal “one mind, many mentalities.” If a theory is not based on the deep structure (one mind), it is probably biased and faces the risk of “infinite regress” (Hwang, 2011b). This is the second issue that will be addressed.

## Toward a culture-inclusive theory of authoritarian orientation

In order to solve the problems abovementioned, formal theories based on universal human deep structure are needed to investigate the substantial theory of authoritarian orientation. More specifically, the authoritarian orientation model needs to be located in a universal theoretical framework in order to be supplemented, modified, or adjusted. Through such a “cultural system approach” (Hwang, 2015a,b), a culture-inclusive theory of authoritarian orientation can be developed that reflects not only the universal mind (one mind) but also the mentality of Chinese authoritarian orientation (one mentality among many).

### The universal models of self and social interaction

In social psychology, two of the most important domains are “relation (social interaction)” and “self.” Hwang (2015a) constructed two theoretical models to represent the universal mechanisms of self and social interaction. One is the *Face and Favor Model* (Hwang, 1987, 2012a) and the other is the *Mandala Model of Self* (Hwang, 2011a,c). According to the principle of “one mind, many mentalities,” both models represent the universal mind and can be used as theoretical frameworks for analyzing a cultural or a sub-cultural system in any given culture (Hwang, 2011b, 2015a,b). In other words, these models can be regarded as formal theories of social psychology.

#### The face and favor model

**Figure 2:**
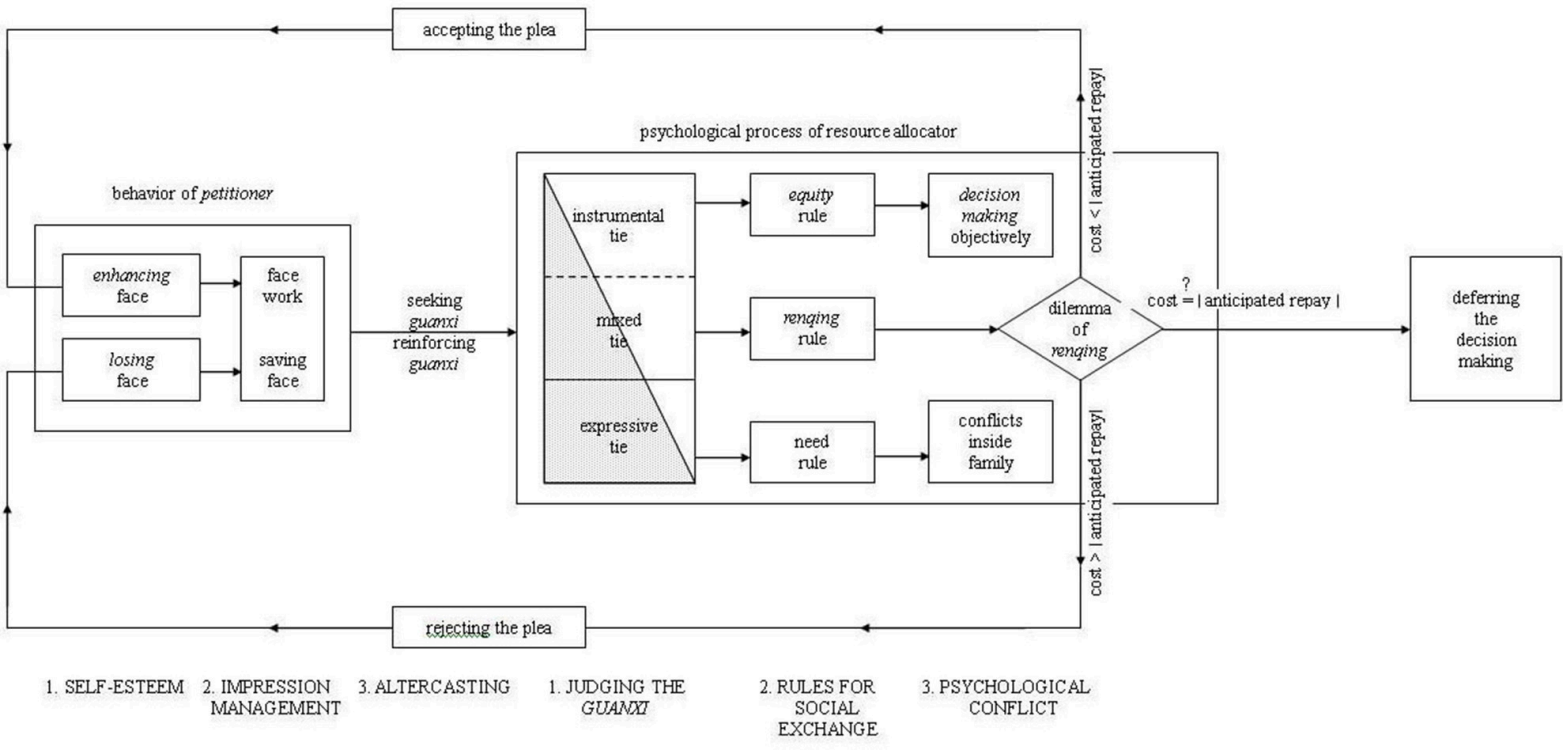
**Face and Favor Model (adapted from Hwang, 1987, p.948)**.

The *Face and Favor Model* depicts the universal mechanism of social interaction. Compared with Fiske's (1992) four elementary forms of relations, this model is based on the deep structure of social relations (Sundararajan, 2015). In Hwang's model, the dyad involved in social interaction comprises “petitioner” and “resource allocator.” When the resource allocator is asked to allocate a social resource to benefit the petitioner, the first thing that the resource allocator would do is make a relationship judgment, that is, to consider “What is the relationship between us?” In Figure 2, relationship is divided into two parts by a diagonal line. The shaded part stands for the affective component of the relationship, while the unshaded part represents the instrumental component. According to the proportion of the affective relative to the instrumental component, interpersonal relationships can be divided into expressive ties, mixed ties, or instrumental ties. Different exchange rules are applicable to these three types of relationships during social interactions. Based on this model, Hwang (2012a) further developed a series of indigenous theories, some of which have been supported by empirical studies (Han et al., 2005; Han and Li, 2008; Chen et al., 2009).

#### The Mandala Model of self

**Figure 3:**
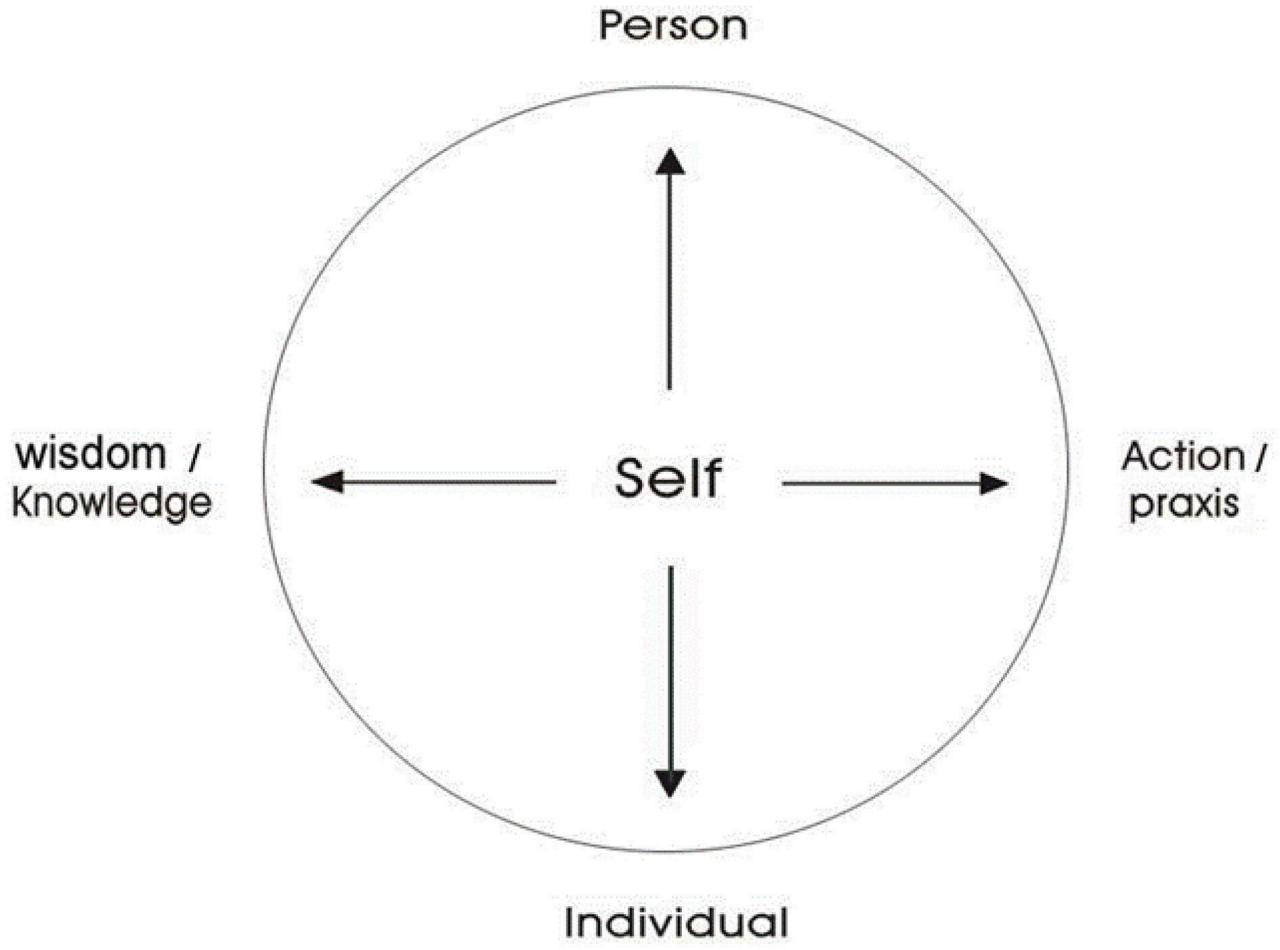
**Mandala Model of Self (adapted from Hwang, 2015a, p.41)**.

Considering the deep structure of self, Hwang (2011a,c) constructed a universal model of self to illustrate the relationship between cultural traditions and an individual's actions. This model has been incorporated into various indigenous researches (Chuang et al., 2014; Hsu et al., 2014; Shiah and Hwang, 2014). In Figure 3, “self” is situated in the center with bi-directional arrows: The top of the vertical arrow points at “person” and the bottom points at “individual.” The right end of the horizontal arrow points at “action” or “praxis,” while the other end points at “wisdom” or “knowledge.” All four concepts are located outside the circle but within the square.

The difference between person, self and individual was pinpointed by Harris (1989). “Person” is a sociological or cultural concept. A person is conceptualized as an agent-in-society who takes a certain standpoint in the social order and plans a series of actions to achieve a particular goal. Appropriate behaviors are defined by every culture, and are endowed with specific meanings and values that can be transmitted to an individual through various channels of socialization. On the other hand, “self” is a psychological concept. It is the locus of experience, able to take various actions in different social contexts and to indulge in self-reflection when hindered from attaining life goals. “Individual” is regarded as members of the human species who are no different from other creatures in the universe.

The self in the *Mandala Model* is able to monitor and to give reasons for his or her own actions. In addition, the self is able to memorize, store and organize various forms of knowledge and make them a well-integrated system of knowledge. However, it is unnecessary for the self to reflect on each of his or her own actions. In everyday life, one intends to, or even has to take some actions when identifying with a particular social role. Over time, the actions become “habitus.” In this model, habitus means an actor's disposition toward praxis or action in a specific social context that enables the actor to carry out the dynamic physical and mental practice within specific socio-cultural orders. In most social situations, an actor may take the action of habitus to engage in social interactions, or to deal with daily affairs.

The “self” exists in a field of forces. When an individual intends to take actions, his or her decisions may be influenced by several forces. When an individual identifies with a particular social role, he or she has to think about how to act as an ideal person in society. On the other hand, an individual is also a biological entity that is motivated by various desires. When these forces are in conflict with each other or when one takes the action of habitus and encounters problems, one may reflect on the actions and search for solutions in the personal stock of knowledge. If the solution is not available, one may take further steps to search for the solution from the social stock of knowledge. Thus, the *Mandala Model* assumes a self with agency and intentionality.

#### Link between the models of self and social interactions

Based on Confucian relationalism, theory development and empirical studies on psychology and social behaviors should take “persons-in-relation” and “person-in-relations” into account (Ho, 1998; Hwang, 2012a). As such, the “self” is inseparable from “relationships” and should be investigated within the relational context. Since the *Face and Favor Model* and the *Mandala Model of Self* represent universal theoretical models for social interaction and self, they should/can be considered together to reinterpret Chien's (2013) emic model.

According to the *Face and Favor Model*, in a social exchange, the allocator should first make a judgment on the relationship. Then, the allocator should follow the need, *renquing* or equity rule to distribute the resources to the petitioner based on their relationship. According to the *Mandala Model*, the allocator in social exchange would follow “benevolence (*ren*),” (favoring people with whom one has a close relationship) which is the criterion of an ideal person in Confucian society. Such an action would become habitus through repetition. If the habitus does not work well, the *Mandala Model* advocates that one may search for a solution from the personal or social stock of knowledge.

Accordingly, if we link the two models of self and social interaction, we are able to explain why Chinese behaviors are usually determined by relations (that is, the relationship determinism) and why relationship determinism can be broken under certain conditions, followed by autonomous actions. Therefore, if the “cultural system approach” (Hwang, 2015a,b) is taken to reinterpret Chien's (2013) model, a culture-inclusive theory of authoritarian orientation will be constructed that achieves the goal of “one mind, many mentalities.” In addition, this theory will be endowed with agency and intentionality.

### Reinterpreting authoritarian orientation in universal models

#### Relationship judgment: authority or not?

The *Face and Favor model* concerns psychological processes and social behaviors of allocators, who own much more resources and are thus more powerful than petitioners. The model of authoritarian orientation deals with Chinese people's psychological processes and social behaviors in vertical social interactions, especially for subordinates. Although the contexts of the two models are not exactly the same, the *Face and Favor Model* does have important implications for developing a culture-inclusive theory of authoritarian orientation. The *Face and Favor Model* indicates that when the resource allocator is asked to allocate a social resource to benefit the petitioner, the first thing that the resource allocator would do is make a relationship judgment. This claim explicitly reflects the fundamental assumption of relationalism, which can also be applied to the theory of authoritarian orientation. Accordingly, we propose that during social interactions, except for judgment of the closeness between the two parties, people would also judge whether the other party is authority or not in order to interact appropriately. The deep structure of Confucian relationalism is organized by proximity (closeness) and hierarchy of relationships. It is the dimension of hierarchy to which the theory of authoritarian orientation pays more attention. Following the authority judgment, the self will be able to decide on the appropriate actions depending on whether or not the other party is an authority.

#### Mandala Model and authoritarian orientation

According to the *Mandala Model*, authority-reverence and authority-obedience belong to aspects of an ideal “person.” They are the cultural demands or ideals for a subordinate in Confucian societies. Authority-dread and authority-dependence are considered components derived unexpectedly from the socialization process; therefore, they are called the “alienation orientation” that emerges to become part of the psychological “self.” They are neither part of an ideal person nor part of a biological “individual.” During an interaction with the authority, Chinese people usually act in accordance with the demand of “person.” Specifically, they would behave respectfully toward the authority and strictly obey the order or request from the authority. In addition, they may feel dread toward the authority and may hope to earn praise and recognition from the authority. As a result from authority-dread and authority-dependence, they may respond in a particular way (Table 1).

The *Mandala Model* claims that, in everyday life, when one identifies with a particular social role (e.g., subordinate role), one would intend to take some actions. As one acts repeatedly, such actions would become a habitus. Similarly, as one identifies with a subordinate role, one would succumb to authoritarian orientation. As one takes authoritarian-oriented actions repeatedly, the actions would become authoritarian-oriented habitus. In this paper, the term “habitus” is used instead of “habit.” Habit is a concept originating from behaviorism, representing the automatic association between an environment or stimulus and a behavior (Wood and Neal, 2007). Although habitus is similar to habit to some extent, habitus entails more socio-cultural meanings. Thus, habitus is more suitable to the cultural perspective we take in this article.

It is worth noting that, based on the *Mandala Model of Self*, if the authoritarian-oriented habitus is not the best strategy or does not work well in some vertical social interactions, other actions or strategies can be taken. Therefore, the revised theory of authoritarian orientation has taken the agency and intentionality of self into consideration. Accordingly, people's interactions with authority are not only determined by habitus but are also relatively flexible.

#### More on “person”: cultural principle of resistance

According to Hwang's (1995) social psychological interpretations, *dāng bú yì zé zhēng zhī* (當 不 義 則 爭 之) should be a cultural principle; if a person commits a wrongful act, then anyone who witnesses this act should fight against it. Based on this principle, even in a vertical relationship, if the superior (authority) violates his or her obligations, those in the subordinate role should voice, resist and even directly revolt. On the cultural level, *dāng bú yì zé zhēng zh*$\bar{ı}$ is a cultural ideal in Confucian society and can be regarded as part of “person.” At the individual level, just as with “authority-reverence” and “authority-obedience,” *dāng bú yì zé zhēng zh*$\bar{ı}$ is defined as a normative belief that a subordinate should resist against the authority violating a superordinate's obligations. Thus, the Confucian cultural system provides a cultural mechanism for disobeying or revolting against the authority, which is endowed with moral legitimization.

It is hypothesized that such an internalized belief will probably influence people's actions. According to the belief, if the superior (authority) violates obligations, those in a subordinate role will resist or even revolt against the authority. The saying “If you're not benevolent, then I'm not righteous” is quite common in Chinese societies. However, once a subordinate takes the action of resistance, the conflict between the two parties becomes explicit. According to the theory on interpersonal harmony and conflict (Huang, 2006), after such an explicit conflict, the relationship will soon enter into superficial harmony (implicit conflict) since explicit conflict is an event that only occurs occasionally. If explicit conflict is too strong, the relationship will probably be broken.

#### Life wisdom: to obey publicly but disobey privately

In addition to “person,” “wisdom” plays an important part in authoritarian orientation theory. Chinese societies can be regarded as systems of “yang-ying duality” (陽 陰 默 認/*yáng y*$\bar{ı}$*n mò rèn*), meaning people may follow the norms or rules in public but violate or resist them in private (Zou, 1998, 2000). Under such a system, “to obey publicly but disobey privately” (陽 奉 陰 違/*yáng fèng y*$\bar{ı}$*n wéi*) is not ethically- or morally-legitimized but a social fact to which everyone acquiesces (Zou, 1998, 2000). On the cultural level, *yáng fèng y*$\bar{ı}$*n wéi* is life wisdom in Chinese societies and can be regarded as part of “wisdom” in the *Mandala Model*. At the individual level, *yáng fèng y*$\bar{ı}$*n wéi* refers to a strategic action, meaning that people say or do one thing in public, but another in private. In a vertical relation, it refers to a subordinate saying or doing one thing when facing the authority, but another in private.

In what conditions will a subordinate take the action of *yáng fèng y*$\bar{ı}$*n wéi*? Everyone has a biological “individual” and a psychological “self” in terms of desires, needs, thoughts and intentions. When these inner voices conflict with the demands of the authority, the default habitus (respecting and obeying responses) may fail to work well due to the fact that the inner voices may not be realized through the existing habitus. If one insists on expressing the inner voice or pursuing personal goals directly, it may not be a wise choice in Chinese culture. However, if one takes the actions of *yáng fèng y*$\bar{ı}$*n wéi*, one can not only avoid direct conflicts with the authority but can also (at least partially) satisfy personal needs or goals privately. A subordinate may resist against the authority violating obligations based on the cultural principle *dāng bú yì zé zhēng zh*$\bar{ı}$. However, it may not be wise to resist under some conditions (e.g., the authority has much more power than the subordinate). As a result, the subordinate may also take *yáng fèng y*$\bar{ı}$*n wéi*. A subordinate is unlikely to disobey or resist against the authority directly until possessing enough resources or power (Huang et al., 2008).

Since *yáng fèng y*$\bar{ı}$*n wéi* can avoid direct conflicts with the authority, the relationship will not be broken but will maintain the state of superficial harmony. Although superficial harmony is not a very good state, it retains the possibility that superficial harmony will become genuine harmony 1 day. As we can see, *yáng fèng y*$\bar{ı}$*n wéi* has positive functions, as life wisdom is highlighted in interpersonal contexts. As a result, “psychosocial homeostasis” can be maintained or achieved (Hsu, 1971; Hwang, 2000). In Western societies, *yáng fèng y*$\bar{ı}$*n wéi* (self-*in*consistency) may be considered insincere; however, it may be considered conducting oneself well (huì zuò rén) in contrast with self-consistency, which is considered immature in Chinese society (Yang C.-F., 1999).

### Preliminary empirical evidences

Until recently, only a few studies have examined Chinese authoritarian orientation. The reason might be that only preliminary conceptualization (Yang, 1993) was available and a first systematic model (Chien, 2013) was only constructed in 2013. A more comprehensive theory is proposed in this paper. Some studies that provided evidence directly or indirectly are briefly introduced below (Lin and Lin, 1999; Huang et al., 2008; Hsu and Huang, 2009; Chien and Huang, 2010, 2015; Liu et al., 2010; Huang and Chu, 2012; Chien, 2013; Chien et al., submitted).

#### The “person”: cultural ideals in the theory

Chien and Huang (2010) investigated the social representations of students' obligations and rights. In a pilot study, undergraduate students were invited to write down a list of students' obligations and rights in an open-ended questionnaire. Their answers were classified into a few items referring to potential role obligations and listed in a checklist. In a follow-up study, participants were asked to check the items that they considered to include students' role obligations. The findings revealed that “reverence for teachers” and “obedience to teachers' instructions” were regarded as students' role obligations from elementary school to college. It indicated that from undergraduates' perspective, a student (a subordinate role) should fulfill the obligation of respecting and obeying the teacher (a superordinate role). In addition, Huang and Chu (2012) utilized a representative sample to investigate the trends of core values in Taiwan. “Obedience to superiors” was found to be highly valued and “respecting superiors” was considered the most important value orientation regarding adequate interpersonal interactions. Taken together, the results supported the cultural construction of a “person” in the theory of authoritarian orientation.

#### The “habitus”: the validation of authority-sensitization

Chien (2013, study 3) investigated Chinese authority-sensitization in social interactions. Participants (mainly undergraduates) were instructed to imagine being “in a social occasion” and then to offer their responses to an authority under in a context. The findings suggested that most participants (more than 80%) were accustomed to verifying whether a person in a higher order of seniority or position (that is, an authority) was nearby on that specific occasion, labeled authority- searching or verification. The results indicated that Chinese people would try to identify the authority at a social occasion, supporting the claim that Chinese people would make hierarchical relationship judgments during social interactions. In addition, when people judged another party to be an authority, they would take authoritarian- oriented actions such as yielding seats, standing up immediately and using honorifics when speaking, to show respect to the authority.

Recently, Chien et al. (submitted, study 1) replicated Chien's study 3 using a sample including nonstudent adults. Following study 1, they investigated the ethical implications and social adjustment of authority-sensitization (study 2). The results showed that the behavioral model of high authority-sensitization, compared with that of low authority-sensitization, was more consistent with the cultural norm for a subordinate role. In addition, those with high authority-sensitization were more likely to have better interpersonal relationship and to be promoted by their superior. Taken together, Chien (2013, study 3) and Chien et al. (submitted) provide evidences for authority-sensitization and its association with positive social adjustment.

#### Resistance to authority violating obligations

According to the culture-inclusive theory of authoritarian orientation, if the superior (authority) violates his or her obligations, those in the subordinate role would undertake the action of resistance. Several studies involving different kinds of vertical relations provide direct or indirect evidences for this proposition (Chien, 2013, study 2; Hsu and Huang, 2009; Liu et al., 2010).

In Hsu and Huang (2009), conflict events among parents and children were classified according to parents' fulfilling or violating obligation. Three kinds of conflict events, “fulfilling positive obligation,” “violating uncompulsory obligation,” and “violating compulsory obligation” were the best predictors of the parent-child relationship after the conflict event. Among the three conflict events, when the conflict was due to parents violating their compulsory obligation, the parent-child relation perceived by children after conflict became negative regardless of whether the prior relationship was good (genuine harmony) or not so good (superficial harmony). Although this study did not measure resistance as a dependent variable, negative relationship would probably drive children to disobey their parents.

Liu et al. (2010) examined the link between supervisor abusive supervision and subordinate supervisor-directed deviance. Hundreds of supervisor-subordinate dyads in private and state-owned companies from mainland China participated in the study. It showed that abusive supervision was positively related to subordinates' revenge cognition toward supervisors, and also positively related to supervisor-directed deviance. In addition, traditionality moderated the above relationships such that they were stronger among low traditionalists than among high ones, while revenge cognition mediated the effect of abusive supervision and the interactive effect of abusive supervision and traditionality on supervisor-directed deviance. Based on Chinese relationalism (Hwang, 2012a), abusive supervision can be considered as supervisors' violation of obligation (see Hsu and Huang, 2009). Therefore, the proposition *dāng bú yì zé zhēng zh*$\bar{ı}$ was supported.

In a scenario experiment, Chien (2013, study 2) examined the impact of advisors fulfilling or violating obligation on advisees' responses to advisors. Graduate students were instructed to read a scenario about the interaction of an advisor and an advisee and offer the advisee's possible responses to the advisor. It showed that when an advisor violated his or her compulsory obligation, the advisee's intentions of respecting the advisor and complying with his/her demands would be significantly decreased. Even after graduation, advisors violating compulsory obligations still had negative effects on these responses and also a destructive effect on the advisee's relationship maintenance intention. Thus, the proposition *dāng bú yì zé zhēng zh*$\bar{ı}$ was again supported in the advisor-advisee relational context.

#### The “wisdom”: evidence for *yáng fèng yīn wéi*

*Yáng fèng y*$\bar{ı}$*n wéi* is ubiquitous in various domains, such as business, law, politics and social interactions (Zou, 2000) but empirical studies have been relatively scarce. An indigenous model on Chinese conflict resolution claims that when a subordinate has conflicts with a superior and knows that it is useless to argue with the other in dominant power, s/he may accept the superior's requests in public, but do his own business in private (Hwang, 1997-1998). The significance of *yáng fèng y*$\bar{ı}$*n wéi* during vertical interaction is made prominent in this model. Based on the philosophy of constructive realism, if a proposition in one theory (microworld) can be translated into the language of another theory, it implies a closer approximation to the truth (Hwang, 2000). The proposition on *yáng fèng y*$\bar{ı}$*n wéi* in the theory of authoritarian orientation can be translated into the language of another theory, so it is close to the truth to some extent.

The transformation process of “ren” (forbearance) was investigated in the context of vertical relations (Huang et al., 2008). Initially, one is obedient during interactions with an authority. As one feels oppressed by the authority, one would forbear (*ren*) and submit to the authority. However, submitting to authority is not the best strategy since long-term self-oppression will lead to psychological maladjustment and unsatisfied personal needs. Eventually, the self will be compartmentalized into public and private self. The public self may submit to authority and the private self may just do what one wants to do. It was also found that one would probably *yáng fèng y*$\bar{ı}$*n wéi* if one has a need or goal which conflicts with the demands of the authority (Li, 1998; Lin and Lin, 1999; Chien and Huang, 2015).

In sum, *yáng fèng y*$\bar{ı}$*n wéi* can not only help one to achieve one's goals but also contribute to a harmonious relationship. *Yáng fèng y*$\bar{ı}$*n wéi* as part of Chinese life wisdom can have positive functions as it is put into practice in an interpersonal context. The above studies provide evidence for *yáng fèng y*$\bar{ı}$*n wéi* although they are qualitative researches (idiographic approach). If we want to know how *yáng fèng y*$\bar{ı}$*n wéi* works and its functions in a larger society, a nomothetic approach can be undertaken in the future.

## Conclusion and general discussion

### Summary

Briefly speaking, the theoretical development of Chinese authoritarian orientation can be divided into three periods. In the first period, Yang (1993) proposed a preliminary classification. In the second period, Chien (2013) took an emic approach to construct the formation process and components of authoritarian orientation from a bottom-up approach. In the third period (this paper), based on “cultural system approach” (Hwang, 2015a,b), this model has been modified and supplemented to be a comprehensive culture-inclusive theory of authoritarian orientation.

The formation of authoritarian orientation begins with the biological “individual” and goes through different orientations, including alienation orientation belonging to “self,” obligation orientation belonging to “person,” and finally habitus that represents people's routines or accustomed actions toward authority. Alienation orientation is a product of the interaction between the Confucian cultural system and society. Although it is not directly related to cultural ideals, it becomes the component of authoritarian orientation with significant impact on people's actions. Obligation orientation is the norm and standard for people's interactions with authorities. It corresponds to the cultural ideals in the Confucian cultural system, and represents the “person” in the *Mandala Model of Self*. When one identifies with the demands of a subordinate role, one may take the actions of respecting and obeying the authority as an ideal person. As time goes by, these actions would be transformed into the habitus of authoritarian orientation.

However, it is worth noting that the theory does not claim that people blindly or consistently respect and obey authority. As we can see, the *dāng bú yì zé zhēng zh*$\bar{ı}$ provides a cultural legitimate basis for resisting or revolting against authority. It is a fact that an authority usually holds more resources, which leads to an imbalance of power structure between the two parties in vertical relationships. As a result, it is difficult to achieve the cultural ideal *dāng bú yì zé zhēng zh*$\bar{ı}$ in practice. Fortunately, the wisdom of *yáng fèng y*$\bar{ı}$*n wéi* from the Chinese social stock of knowledge has offered another option whereby people can preserve their intentions or satisfy their own needs without disobeying the demands of an authority. As long as the subordinate has accumulated a certain level of capabilities and resources, s/he can decide whether or not to fight against the authority.

### Advantages of the revised theory

Compared with the original model of authoritarian orientation constructed by Chien (2013), the revised theory supplements the old one while being more inspiring. Although the old model did not deny the roles of agency and intentionality, it failed to provide a proper position and clear illustration for them.

The revised theory takes Hwang's universal theories of self and social interaction as meta-theories (Hwang, 1987, 2011a,c), especially the *Mandala Model of Self*. The *Mandala Model of Self* can be used to illustrate the relationship between cultural traditions and individual actions; it also advocates that the “self” exists in a field of forces and may be influenced by several forces. Therefore, the relationship between cultural values and individual actions is not deterministic. For example, when interacting with the authority, the “person” and “habitus” of authoritarian orientation would guide people's actions. However, their intentions also play an important role in directing behaviors, especially when habitus cannot be applied. Therefore, authoritarian orientation can be flexibly regulated in specific situations if needed. This is the key difference from the trait approach advocated by the authoritarian personality.

In addition, the original model of authoritarian orientation can illustrate why Chinese people revere and obey authority, reflecting conformity to Confucian cultural ideals, although it cannot explain why Chinese people would disobey or rebel against authority in specific situations. In this paper, such a possibility has been considered into the revised theory. Notwithstanding, if the authority possesses too much power or if the subordinate does not want an open break in the relationship with the authority, s/he would probably take the strategic actions of *yáng fèng y*$\bar{ı}$*n wéi*, which reflects the flexibility of the authoritarian orientation. Thus, the revised model can explain when Chinese people obey and disobey, showing a broader coverage than the original model's.

### Robustness of authoritarian orientation

The mode of authoritarian orientation reflects the cultural “mentality” of interactions in vertical relationships for Chinese people. However, in the age of globalization, to what extent will the cultural traditions as well as authoritarian orientation be preserved? Li (2002), an indigenous psychologist, indicated that among social orientations, the “relationship” orientation would be preserved permanently due to its evolutional and biological basis, while “familistic,” “authoritarian,” and “other” orientations would probably gradually disappear due to the effects of industrialization, technological progress and urbanization. However, even under the influence of Western culture in the East, the authoritarian orientation may probably still remain robust, based on the perspective of structuralism and the cultural mechanism provided by Chinese wisdom.

From the perspective of structuralism, human social interactions can be divided into four elementary forms: communal sharing, authority ranking, equality matching and market pricing (Fiske, 1992). These forms exist throughout human societies (Fiske, 1992) and are also deep structures of social interactions (Hwang, 2012a). Among the four forms of interactions, authoritarian orientation directly corresponds to authority ranking. Furthermore, Confucian relationalism is governed by the principle of “favoring the intimate” and “respecting the superior”; it is the cultural deep structure of Confucian ethics and a synchronic structure, which exists all the time (Hwang, 2012a). As we can see, Chinese authoritarian orientation can be regarded as a cultural mentality that is based on the universal human deep structure of social interactions, as well as the cultural principle of respecting the superior. Thus, authoritarian orientation would be preserved as well.

In addition, Chinese societies can be regarded as systems of “yang-ying duality.” Under such systems, *yáng fèng y*$\bar{ı}$*n wéi* is a common action strategy and also a social fact to which everyone acquiesces (Zou, 1998, 2000). It enables a subordinate to preserve autonomy and intention without publicly resisting the authority. This might be another reason why authoritarian orientation is well-preserved today.

### A proposal for future directions

As a newly developed theory, a series of studies can be conducted to examine the propositions and hypotheses derived from the authoritarian orientation theory. First, a longitudinal research is needed to investigate the formation process of authoritarian orientation. The process herein was constructed through a qualitative study and was grounded in data (Chien, 2013). Although inspiring, researchers can conduct more studies with developmental research methods.

Authoritarian orientation can be investigated from the perspective of personality (trait) and that of social interaction (Yang, 1993). For the components of authoritarian orientation, no appropriate measurement was developed. Hence, a suitable measurement can be constructed in the future. Researchers can use a well-developed measurement to investigate various subordinate-superior interactions. For example, in regard to a teacher-student relationship, how does authoritarian orientation affect students' interaction with their teachers? How does authoritarian orientation influence students' learning outcome? How does authoritarian orientation interact with various social situations?

The theory has proposed that during social interactions with authority, the authoritarian-oriented actions (habitus) will be exhibited by a subordinate. In general, the habitus can be considered as default responses to authority. However, under some conditions illustrated in Sections More on “Person”: Cultural Principle of Resistance and Life Wisdom: To Obey Publicly but Disobey Privately, instead of the authoritarian-oriented habitus, the actions of *yáng fèng y*$\bar{ı}$*n wéi* or resistance based on *dāng bú yì zé zhēng zh*$\bar{ı}$ will be undertaken. This means that Chinese people do not always respect or obey the authority. Actually, the habitus can be restrained if necessary. More solid and direct support for the triggering conditions of *yáng fèng y*$\bar{ı}$*n wéi* and *dāng bú yì zé zhēng zh*$\bar{ı}$ are needed although some evidence does exist.

Furthermore, is authoritarian orientation helpful or harmful to psychological and social adaptation? It is hypothesized that authoritarian orientation emerges from the long-term interaction between an individual and authority under the cultural context of familism. Authoritarian orientation meets Confucian cultural demands in Chinese societies. The cultural fit may contribute to psychological adaptation as well as psychological well-being (Lu, 2006). Based on the assumption of relationalism, authoritarian orientation should be investigated in relational contexts. People may interact with different authority figures in different vertical relationships. Hence, the relationship between authoritarian orientation and psycho-social adaptations may depend on specific relational contexts or the experiences of interactions between the two parties. As a result, the social interaction approach might be more appropriate than the trait approach for empirical researches.

The robustness of authoritarian orientation does not mean that it can never be changed. Authoritarian orientation has been constructed as a systematic model with various components. Thus, different components can be investigated independently. For example, Taiwan, a Chinese society, has gone through modernization and democratization. The values of democracy and egalitarianism emerged along with autonomy. They would be in conflict with authority-obedience. Moreover, authority-dread would be undesirable as it entails a negative component. Therefore, authority-obedience and authority-dread might evolve at the societal level. In sum, the evolution of authoritarian orientation involves lots of complicated factors. Further investigations will be needed.

## Conclusion

This paper details the construction of the authoritarian orientation model as a systematic theoretical framework. Unlike the Western approach of authoritarian personality, this model takes a different approach to examining Chinese authoritarian orientation. Compared with Yang's (1993) preliminary conceptualization, the new model enables us to measure the relevant constructs more easily; thus, a series of future studies can be conducted. In addition to indigenous empirical studies, the construction of indigenous theories (culture-inclusive theories) is even more important for the development of indigenous psychology because only new theories, not empirical studies, can compete with and replace existing mainstream theories (Hwang, 2011c). The progress of the authoritarian orientation model has demonstrated a paradigm for the construction of indigenous psychological theories.

## Author contributions

CC: Substantial contributions to the conception of the work and drafting the work. Final approval of the version to be published. Agreement to be accountable for all aspects of the work.

### Conflict of interest statement

The author declares that the research was conducted in the absence of any commercial or financial relationships that could be construed as a potential conflict of interest.
